# Value of simple clinical parameters to predict insulin resistance among newly diagnosed patients with type 2 diabetes in limited resource settings

**DOI:** 10.1371/journal.pone.0248469

**Published:** 2021-03-31

**Authors:** Keddagoda Gamage Piyumi Wasana, Anoja Priyadarshani Attanayake, Thilak Priyantha Weerarathna, Kamani Ayoma Perera Wijewardana Jayatilaka

**Affiliations:** 1 Department of Biochemistry, Faculty of Medicine, University of Ruhuna, Galle, Sri Lanka; 2 Department of Medicine, Faculty of Medicine, University of Ruhuna, Galle, Sri Lanka; Laval University, CANADA

## Abstract

**Background:**

Insulin resistance (IR) has been considered as a therapeutic target in the management of type 2 diabetes mellitus (T2DM). Readily available, simple and low cost measures to identify individuals with IR is of utmost importance for clinicians to plan optimal management strategies. Research on the associations between surrogate markers of IR and routine clinical and lipid parameters have not been carried out in Sri Lanka, a developing country with rising burden of T2DM with inadequate resources. Therefore, we aimed to study the utility of readily available clinical parameters such as age, body mass index (BMI), waist circumference (WC) and triglyceride to high density lipoprotein cholesterol ratio (TG/HDL-C) in the fasting lipid profile in predicting IR in a cohort of patients with newly diagnosed T2DM in Sri Lanka.

**Methods and findings:**

We conducted a community based cross sectional study involving of 147 patients (age 30–60 years) with newly diagnosed T2DM in a suburban locality in Galle district, Sri Lanka. Data on age, BMI, WC, fasting plasma glucose (FPG) concentration, fasting insulin concentration and serum lipid profile were collected from each subject. The indirect IR indices namely homeostasis model assessment (HOMA), quantitative insulin sensitivity check index (QUICKI) and McAuley index (MCA) were estimated. Both clinical and biochemical parameters across the lowest and the highest fasting insulin quartiles were compared using independent sample t-test. Linear correlation analysis was performed to assess the correlation between selected clinical parameters and indirect IR indices. The area under the receiver operating characteristic (ROC) curve was obtained to calculate optimal cut-off values for the clinical markers to differentiate IR. BMI (p<0.001) and WC (p = 0.01) were significantly increased whereas age (p = 0.06) was decreased and TG/HDL-C (p = 0.28) was increased across the insulin quartiles. BMI and WC were significantly correlated (p<0.05) with HOMA, QUICKI and MCA. Out of the clinical parameters, age showed a borderline significant correlation with QUICKI and TG/HDL-C showed a significant correlation only with MCA. The area under ROC of BMI was 0.728 (95% CI 0.648–0.809; p<0.001) and for WC, it was 0.646 (95% CI 0.559–0.734; p = 0.003). The optimized cut-off value for BMI and WC were 24.91 kg/m^2^ and 81.5 cm respectively to differentiate the patients with IR or ID. Study limitations include small sample size due to recruitment of patients only from a limited geographical locality of the country and not totally excluding of the possibility of inclusion of some patients with slowly progressive type 1 DM or Latent onset diabetes of adulthood from the study population.

**Conclusions:**

The results revealed that there was a significant positive correlation between BMI, WC and HOMA while a significant negative correlation with QUICKI and MCA among the cohort of patients with newly diagnosed T2DM. The cut-off values of BMI and WC as 24.91 kg/m^2^ and 81.5 cm respectively could be used as simple clinical parameters to identify IR in newly diagnosed patients with T2DM. Our results could be beneficial in rational decision making in the management of newly diagnosed patients with T2DM in limited resource settings.

## Introduction

Diabetes mellitus (DM) is a major health burden in the world [1]. Type 2 diabetes mellitus (T2DM) is the most common type, accounting for nearly 90% of all cases of diabetes [2]. T2DM is characterized by insulin deficiency (ID) or insulin resistance (IR) or combination of both [2]. IR is a pathological condition characterized by inadequate physiological response of peripheral tissues to circulating insulin [3]. There are many causes of IR, however, the exact underlying mechanism has not been fully elucidated. Risk factors for IR include obesity, sedentary lifestyle, family history of DM, various endocrine disorders, and certain medications. IR is often found in individuals with visceral adiposity, hypertension, hyperglycemia, and dyslipidemia involving elevated triglycerides (TG), low-density lipoprotein cholesterol (LDL-C), and decreased high-density lipoprotein cholesterol (HDL-C) levels [4]. The link between IR and T2DM has been well established [5]. ID, the inability of pancreatic β-cells to secrete sufficient insulin in response to hyperglycemia, is the transition state from IR to T2DM [6].

There are multiple ways to measure IR in different settings. Hyperinsulinemic euglycemic clamp method is considered as the gold standard for direct assessment of IR [7]. Other than the gold standard, intravenous glucose tolerance test and minimal model approximation of the metabolism of glucose, insulin suppression test are the alternative methods available to assess IR directly [8]. However, the assessment of IR using these direct methods are reserved only for intensive physiological studies on small number of samples due to their high cost, delay in analysis, technical requirements etc. Hence, the clinical utility of these measures is limited in routine clinical practice and in large population-based studies. Due to intricate nature of the direct methods and their potential risk of hypoglycemia in some patients, substitute approaches have been developed to simplify the assessment of IR over past few years. Indirect surrogate indices such as homeostasis model assessment (HOMA), quantitative insulin sensitivity check index (QUICKI) and McAuley index (MCA) are some of the indirect tools introduced to measure IR [9–11]. The HOMA model was developed to quantify IR using the concentration of fasting plasma glucose (FPG) and fasting insulin. QUICKI model is simply a variation of HOMA equations by transforming the data considering both the logarithm and the reciprocal of the glucose-insulin product, thus slightly slanting the distribution of fasting insulin values [10]. Background of the MCA index differs from HOMA and QUICKI as it estimates IR using fasting insulin and TG concentration [11]. In contrast to the cumbersome and expensive clamp method, HOMA, QUICKI and MCA indices provide convenient and inexpensive means of estimation of IR [12]. Validation of all these indirect indices of IR with the gold standard clamp method has been reported in several research studies. [10, 13, 14].

There are several practical limitations associated with aforementioned IR indices as these are based on biochemical investigations which require laboratory facilities, quality control measures, necessity of multiple blood sampling etc. Therefore, a simple, readily available, low-cost measures to identify individuals with IR is important for clinical settings with limited resources to plan out optimal management strategies for patients with T2DM. These selected clinical parameters have been tested in previous studies to a limited extent, particularly with a single surrogate index of IR [15, 16]. However extensive research is still needed in this aspect especially on their applicability in settings with limited resources. Knowledge on the association of these simple clinical parameters with IR would be beneficial to clinicians in implementing the most appropriate management protocols such as selecting a suitable oral hypoglycemic agent for newly diagnosed patients with T2DM. Studies on association between the surrogate markers of IR and routine clinical and lipid parameters have not been carried out in Sri Lanka, a developing country with a rising burden of T2DM with limited resources. Therefore, we aimed to study the utility of readily available clinical parameters such as age, body mass index (BMI), waist circumference (WC) and TG/HDL-C in the fasting lipid profile in predicting IR in a cohort of patients with newly diagnosed T2DM in Sri Lanka.

## Materials and method

### Ethical considerations

This study was reviewed and approved by the Ethical Review Commitee, Faculty of Medicine, University of Ruhuna, Sri Lanka (14.06.2017:3.9). Informed written consent was obtained prior to the collection of data and blood sample from study participants.

### Study subjects

This was a community based cross-sectional study conducted in suburban locality in Galle district, Southern, Sri Lanka during January 2018 to December 2019. The study population consisted of 147 newly diagnosed patients with T2DM belonging to age group of 30–60 years. Subjects with known renal, liver, cardiac, respiratory, other chronic or acute diseases, thyroid disorder, psychiatric problems as well as pregnant women and subjects who were on antidiabetic or antihyperlipidemic drugs were excluded from the study.

### Laboratory and clinical measurements

All recruited participants were invited to visit Faculty of Medicine, University of Ruhuna, Sri Lanka. Participants were asked to fast for 8–10 hours before the blood investigations. The selected participants who consented for the study were subjected to FPG, fasting insulin measurements and to serum lipid profile. Collection of blood samples was carried out by a qualified phlebotomist using the standard protocols. Blood collected tubes were centrifuged on site within an hour. Plasma/serum was immediately removed from the tubes and stored at -80°C until analysis. FPG concentration was determined by enzymatic glucose oxidase method using a spectrophotometric assay kit [17]. Serum concentration of fasting insulin was estimated by enzyme linked immunosorbent assay (ELISA) method [18]. Estimation of TG, TC and HDL-C were carried out using spectrophotometric assay kits [19–21]. Low density lipoprotein cholesterol (LDL-C) and very low-density lipoprotein (VLDL-C) were calculated using the Friedewald equation [22]. All laboratory tests were quality controlled and biochemical estimations were done in duplicates. Patients with FPG concentration ≥ 126 mg/dL and/or HbA_1C_ ≥ 6.3% were diagnosed with DM [23].

IR indices were calculated using following equations [9–11].

$$HOMA=\frac{insulin\;\left(mIU/L\right)\times FPG\;\left(mmol/L\right)}{22.5}$$

$$QUICKI=\frac{1}{Log\;insulin\;\left(mIU/L\right)+log\;FPG\;\left(mg/dL\right)}$$

MCA=exp[2.63–0.28ln(insulininmIU/L)–0.31ln(TGinmmol/L)]

In addition, TG/HDL-C ratio was calculated.

Patients were considered as IR when HOMA ≥2.6, QUICKI ≤0.33 and MCA ≤5.8 [11, 24].

The clinical data including age, gender, height, weight and WC were obtained from the enrolled study subjects. Weight was recorded to the nearest 0.5 kg and height was measured to the position without shoes using a height bar. WC was recored using a flexible measuring tape. BMI of study subjects was calculated as weight (kg) devided by height squared (m^2^).

### Statistical analysis

The continuous data were expressed as mean ± standard deviation (SD). All the categorical data were represented as percentages. After the normality of the variables was checked using the Kolmogorov-Smirnov test, independent sample t-test was used to compare both clinical and biochemical parameters across the lowest and highest fasting insulin quartiles of the study subjects. The correlation between clinical parameters and indirect IR indices were assessed through the linear correlation analysis. Receiver operating characteristic (ROC) curves were generated for clinical parameters which were significantly correlated with all IR indices as predictors of IR. Patients were considered as IR by combination of all three indices of HOMA, QUICKI and MCA. The optimal cut-off values for IR prediction of the clinical parameters were determined by using Youden index (maximum [sensitivity + specificity − 1]) [25]. *p* ≤ 0.05 was considered as significant difference at all the cases. All the data were analyzed using SPSS software version 25.

## Results

The total number of enrolled participants was 147 comprising of 61.9% females and 38.1% of males. The clinical and biochemical cherecteristics of the study subjects are summerized in Table 1.

**Table 1:** Clinical and biochemical parameters of the study subjects.

Parameter	Mean ± SD
Age (years)	48.37 ± 7.10
BMI (kg/m^2^)	25.14 ± 3.96
WC (cm)	88.82 ± 9.00
TC (mg/dL)	189.85 ± 44.07
TG (mg/dL)	95.16 ± 54.77
HDL-C (mg/dL)	50.88 ± 22.89
LDL-C (mg/dL)	119.94 ± 47.32
VLDL-C (mg/dL)	19.03 ± 10.95
FPG (mg/dL)	121.04 ± 30.17
Fasting insulin (mIU/L)	18.15 ± 10.81
HOMA	5.51 ± 3.85
QUICKI	0.31 ± 0.04
MCA	6.92 ± 1.95
TG/HDL-C ratio	2.22 ± 1.56

BMI, body mass index; WC, waist circumference; TC, total cholesterol; TG, triglyceride; HDL-C, high density lipoprotein cholesterol; LDL-C, low density lipoprotein cholesterol; VLDL-C, very low density lipoprotein cholesterol; FPG, fasting plasma glucose; HOMA, homeostasis model assessment; QUICKI, quantitative insulin sensitivity check index; MCA, McAuley index.

The analyzed clinical and biochemical parameters across the lowest and highest fasting insulin quartiles are summarized in Table 2.

**Table 2:** Clinical and biochemical characteristics across the lowest (1^st^) and highest (4^th^) fasting insulin quartiles.

Parameter	1^st^ quartile (mean ± SD)	4^th^ quartile (mean ± SD)	*p* value
Age (years)	50.51 ± 5.89	47.57 ± 7.45	0.06
BMI (kg/m^2^)	22.34 ± 3.17	26.38 ± 3.28	0.00
WC (cm)	85.08 ± 8.23	89.62 ± 7.89	0.01
FPG	119.87 ± 30.71	124.74 ± 36.67	0.53
Fasting insulin (mIU/L)	5.62 ± 1.51	32.34 ± 2.81	0.00
TC (mg/dL)	182.94 ± 48.03	194.63 ± 42.64	0.27
TG (mg/dL)	82.08 ± 42.75	108.31 ± 79.79	0.08
HDL-C (mg/dL)	52.19 ± 22.50	50.73 ± 21.98	0.77
LDL-C (mg/dL)	114.33 ± 48.43	122.24 ± 47.07	0.47
VLDL-C (mg/dL)	16.42 ± 8.55	21.66 ± 15.96	0.08
TG/HDL-C	1.98 ± 1.43	2.42 ± 1.93	0.28

BMI, body mass index; WC, waist circumference; FPG, fasting plasma glucose; TC, total cholesterol; TG, triglyceride; HDL-C, high density lipoprotein cholesterol; LDL-C, low density lipoprotein cholesterol; VLDL-C, very low density lipoprotein cholesterol.

**Fig 1:**
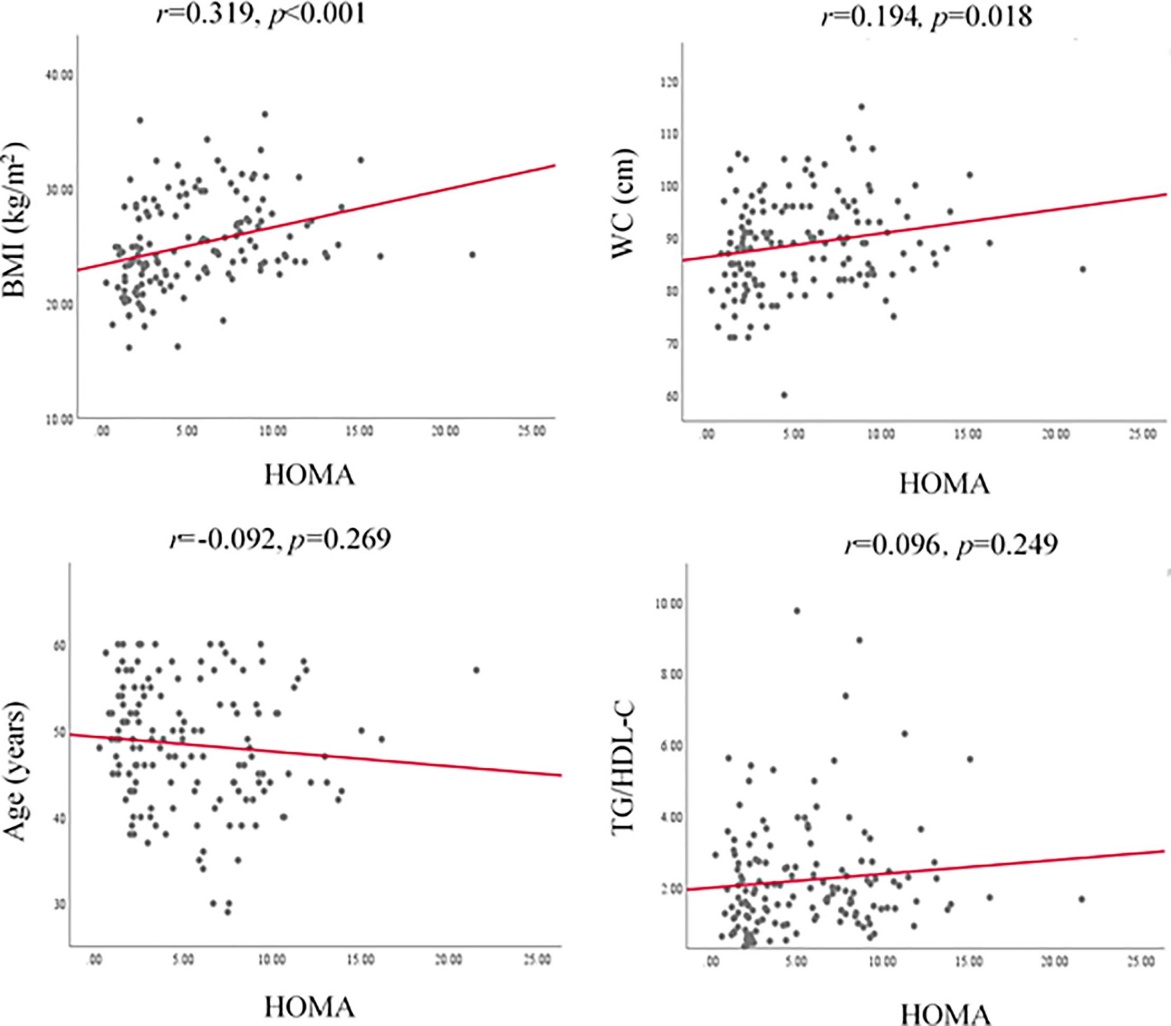
Correlation of HOMA index with BMI, WC, age and TG/HDL-C. BMI, body mass index; WC, waist circumference; TG, triglyceride; HDL-C, high density lipoprotein cholesterol; HOMA, homeostasis model assessment.

**Fig 2:**
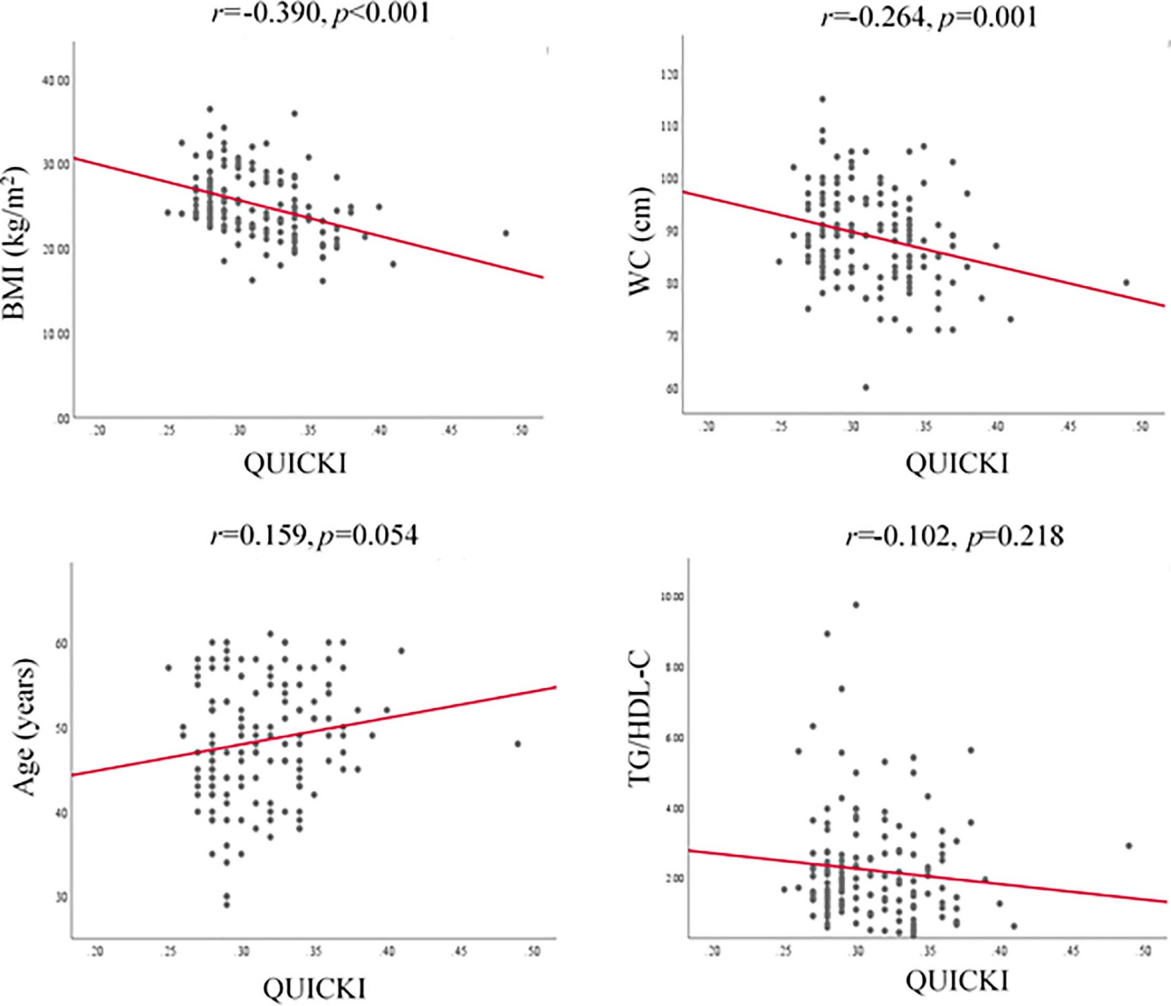
Correlation of QUICKI index with BMI, WC, age and TG/HDL-C. BMI, body mass index; WC, waist circumference; TG, triglyceride; HDL-C, high density lipoprotein cholesterol; QUICKI, quantitative insulin sensitivity check index.

**Fig 3:**
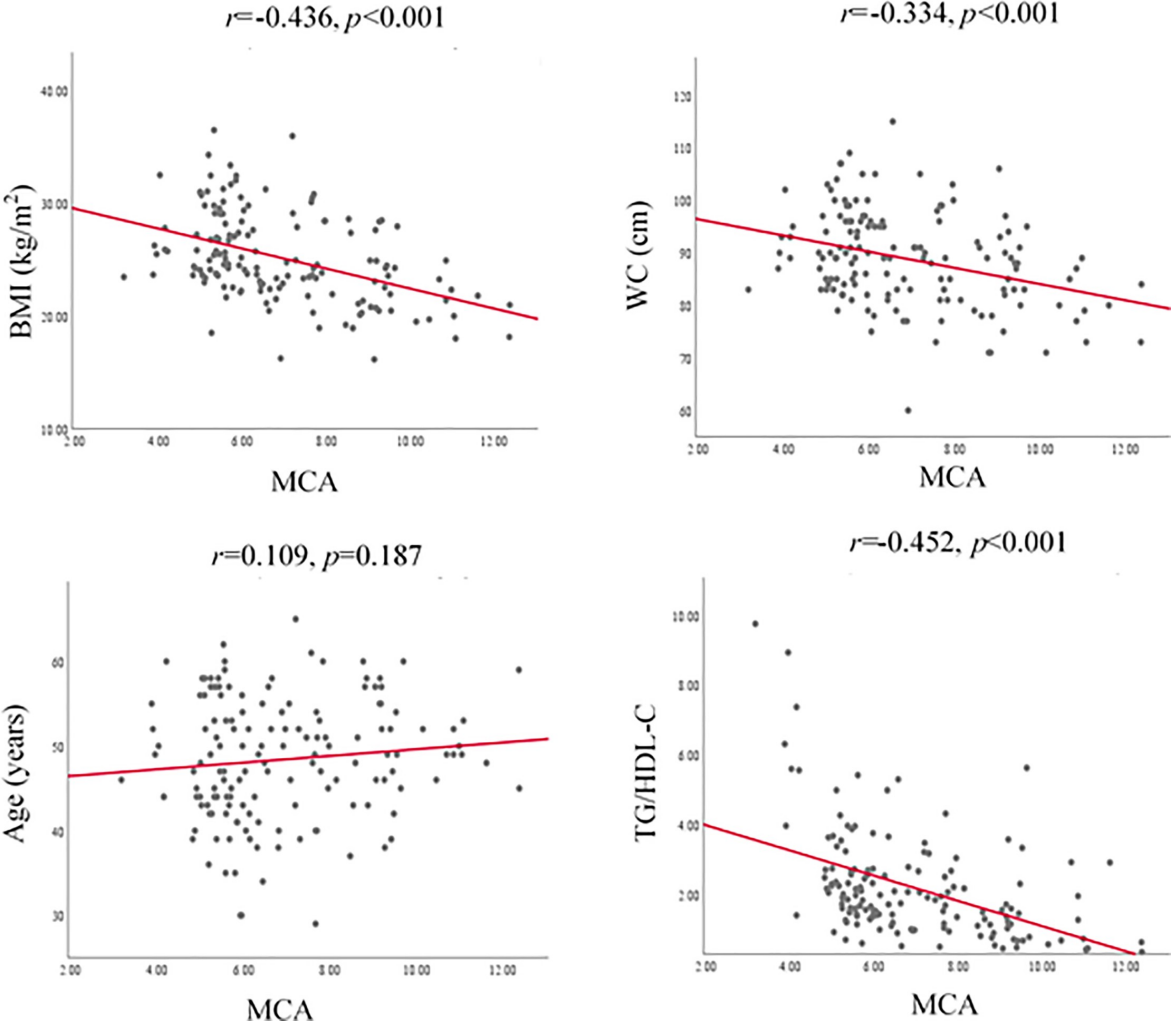
Correlation of MCA index with BMI, WC, age and TG/HDL-C. BMI, body mass index; WC, waist circumference; TG, triglyceride; HDL-C, high density lipoprotein cholesterol; MCA, McAuley index.

Pearson correlation coefficients relating HOMA, QUICKI and MCA indices with BMI, WC, age and TG/HDL-C ratio are represented in Figs 1–3 respectively.

**Fig 4:**
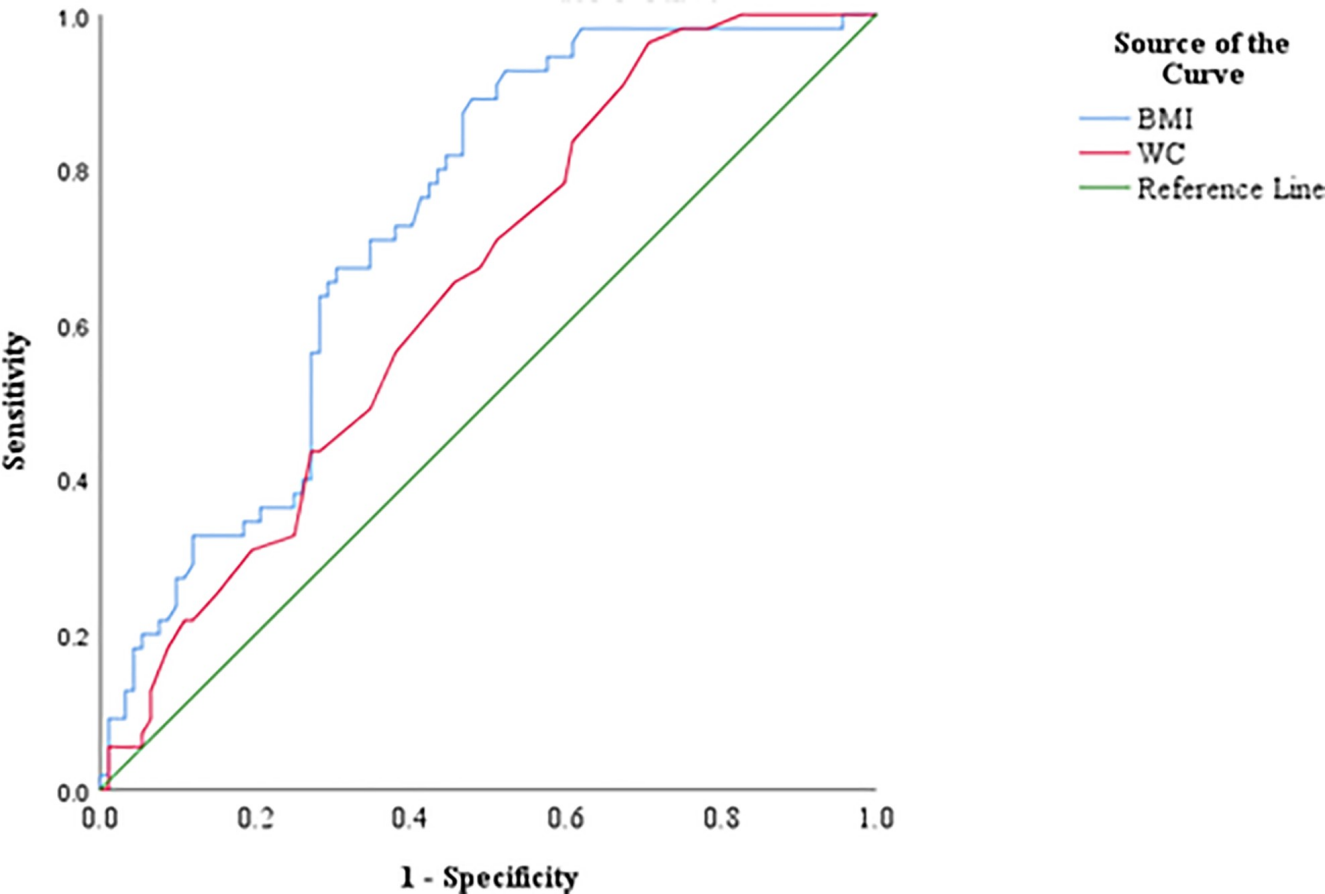
ROC curve for BMI and WC as predictors of IR. BMI, body mass index; WC, waist circumference.

Accordingly, HOMA index showed positive and significant correlation with BMI and WC while QUICKI and MCA showed negative and significant correlations with BMI and WC. Fig 4 shows the ROC curve of BMI and WC as the predictors of IR. The area under the ROC curve (AUC) of ability of BMI (0.728±0.041, p<0.001) and WC (0.646 ± 0.045, p = 0.003) to predict IR was significant. The optimal cut-off value of BMI was 24.91 kg/m^2^ (sensitivity 67.3%, specificity 69.6%) and for WC, cut-off value was 81.5 cm (sensitivity 96.4%, specificity 29.3%) to predict IR (Table 3).

**Table 3:** The AUC, sensitivity, specificity and the optimal cut-off value of BMI and WC in the prediction of IR.

Clinical parameter	AUC (95% CI)	Sensitivity (%)	Specificity (%)	Cut-off value
BMI (kg/m^2^)	0.728 (0.648–0.809)	67.3	69.6	24.91
WC (cm)	0.646 (0.559–0.734)	96.4	29.3	81.5

BMI, body mass index; WC, waist circumference.

## Discussion

Advancing age, increasing BMI and WC are well-known risk factors of T2DM. Results of the present study revealed that BMI and WC are significantly increased across the quartiles of fasting insulin. The BMI and WC were positively correlated with HOMA while BMI and WC were negatively correlated with QUICKI and MCA (p<0.05). In fact, the surrogate markers of IR; HOMA, QUICKI and MCA are not unanimous due to the differences in their compositions of the formulae [9–11]. This fact leads to rise in IR with increasing of HOMA index while QUICKI and MCA indices show a decrement with rising IR [11, 24]. Therefore, the clinical parameters showed a positive correlation with HOMA index and a negative correlation with QUICKI and MCA in the present study. Relative body size and obesity reflected by BMI and WC play a role in the presence of IR. The underlying potential mechanism for positive correlation between BMI and WC with IR may be due to impairment of adipogenesis and reduction in lipogenesis in subcutaneous fat resulting a high rate of deposition of fat and larger sizes of visceral adipocytes [26]. Deposition of visceral fat is associated with an enhanced secretion of inflammatory cytokines which deregulates insulin signaling pathways that have a negative impact on IR [27]. Further, excess nutrients may enhance exposure of cells and tissues to high concentration of fatty acids that inhibit insulin signaling pathways through the activation of protein kinase C in the liver and muscle. Excess accumulation of lipids in individuals having high BMI and WC could trigger an increase in reactive oxygen species (ROS) generated from the mitochondrial cell oxidation [28]. An exposure of cells to excess ROS leads to an activation of serine or threonine kinase that inhibit insulin signaling pathways. All these mechanisms result IR in parallel to an increase in BMI and WC. Significant correlations between BMI and WC with IR measured by HOMA, QUICKI and MCA indices have been reported previously to a limited extent [15, 29, 30], however the selected indices; HOMA, QUICKI and MCA were not reported collectively in a single study on newly diagnosed T2DM patients. Hence, this is the first report of correlation analysis of the selected clinical parameters of BMI, WC, age and TG/HDL-C ratio with indices of HOMA, QUICKI and MCA on newly diagnosed patients with T2DM.

Even though the age of the patients was not significantly increased (p = 0.06) across the insulin quartiles, the results showed that younger patients with newly diagnosed T2DM are more likely to be hyperinsulinemic compared to older ones. This fact was further supported by liner correlation analysis as it was implicated a non-significant negative correlation between fasting insulin concentration and age of the study population (p = 0.074, r = -0.148). A negative association of age with random plasma insulin level has previously reported by Bryhni et al. [31]. Probable underlying mechanisms for having lower plasma insulin levels with aging could be explained by several factors including reduction of β-cell mass and/or malfunctions due to accumulation of amyloid proteins in the islet cells, lipotoxicity, the actions of circulatory adipocytokines, or a diminished effect of incretin hormones in the elderly. Another explanation for older patients having low fasting insulin level could be due to the fact that they preferentially take small meals or have composition to trigger less insulin release in response to a meal, or have slower rates of gastric emptying and delayed nutrient absorption [32]. Consequences of hyperinsulinemia at the time of diagnosis in younger patients with T2DM can affect the natural history of the disease. Continued high insulin secretion over many years can exhaust pancreatic β cells and deplete endogenous insulin altogether, necessitating early exogenous insulin therapy to control hyperglycemia. The observation that younger onset T2DM requiring exogenous insulin earlier than those develop T2DM at middle age or later in some studies substantiates our findings [33].

Even though previous studies have revealed that TG/HDL-C ratio as a good surrogate marker to predict IR in obese children or youth [34, 35], in this study, we found it is not a significant predictor of IR in patients with newly diagnosed T2DM. However, we found that, the ratio of TG/HDL-C was increased (p = 0.28) across the insulin quartile suggesting its positive association with IR. The metabolites of TG such as free fatty acids, diacylglycerol and etc. could interfere with insulin signaling pathway via activation of several kinases which repress insulin receptor and tyrosine phosphorylation of insulin receptor substrates, with an aim with inducing of IR [36–38]. HDL-C suppress the inducible nitric oxide and fatty acid synthase and it causes an induction of IR. [39]. Accordingly, TG and HDL-C could induce IR through multiple mechanisms.

Results of the correlation analysis revealed BMI and WC are good clinical markers to predict IR in newly diagnosed patients with T2DM. However, age showed a borderline significant correlation only with QUICKI (r = 0.159, p = 0.054) and TG/HDL-C ratio was only significantly correlated with MCA (r = -0.452, p<0.001). As it was important to define cut-off values for BMI and WC in patients with newly diagnosed T2DM to identify IR, ROC curves were drawn. According to the ROC curve analysis results, cut-off value for BMI was 24.91 kg/m^2^ with 67.3% sensitivity and 69.6% specificity and for WC measurement, cut-off value was 81.5 cm with 96.4% sensitivity and 29.3% specificity. The AUC value for BMI (0.728) in ROC analysis unveiled that BMI>24 kg/m^2^ could be used as an acceptable clinical parameter to differentiate IR or ID in newly diagnosed patients with T2DM. Even though it is recommended that AUC value of 0.7 to 0.8 as acceptable and 0.5 is not acceptable to discriminate of patients who are having or not having the disease condition [40], AUC value of WC in this study (0.646) indicates its ability to differentiate patients with IR or ID to a limited extent. Further, when the WC values of men and women in the present study are considered separately, the men and women could be differentiated as IR or ID with the cut-off value of 83.5 cm (95.2% sensitivity and 34.3% specificity) and 81.5 cm (94.1% sensitivity and 29.8% specificity) respectively. Similar observation with BMI and WC as good predictors to determine IR has been reported previously for middle-aged and elderly Taiwanese [15].

Findings of this study empowers clinicians to implement most appropriate management strategy for patients with newly diagnosed T2DM with IR based on their age, BMI and WC or TG / HDL ratio in the lipid profile without any expensive testing for IR. These strategies would include targeting weight loss, reducing central obesity and more importantly preferential selection of insulin sensitizing agents such as metformin or pioglitazone to ameliorate IR in newly diagnosed patients with higher BMI and WC. Inadvertent use of insulin or insulin secretagogues such as sulphonylureas in patient with IR would make them more hyperinsulinemic and augment many adverse consequences. For an example, if those with higher insulin levels with IR were started on insulin secretagogues, they would require early exogenous insulin therapy due to the iatrogenic exhaustion of pancreatic insulin reserves. Our findings of patients with lower BMI and WC having reduced insulin levels would also enable clinicians to be more selective in requesting expensive tests for the individuals with newly diagnosed diabetes due to ID states such as slowly progressive type 1 DM (T1DM).

Therefore, we believe that findings of this study have significant clinical implications in the initial categorization of newly diagnosed patients with diabetes and recommending the most appropriate and optimal management strategies for them especially in limited resource settings.

The main strength of the present study is that the recruitment of previously healthy, newly diagnosed patients who are clinically having T2DM. Therefore, insulin level, plasma glucose concentration and serum lipid profile of patients were not affected by any lifestyle modifications or pharmacological interventions. Due to financial constraints, we couldn’t measure the antibodies which are known to be present in patients with less frequent subtypes of diabetes such as type 1 diabetes and Latent onset diabetes of adulthood (LADA). Hence, we couldn’t totally exclude the possibility of inclusion of some patients with slowly progressive T1DM or LADA in the subset of patients in the lowest quartile of insulin in this study. Relatively small sample size and recruitment of patients only from a limited geographical locality of the country are the other associated limitations of the present study. Therefore, it is important to extend the study to a larger study sample with geographically wider representation. Further, it is important to consider several other risk factors associated with DM, to broaden the output of the study.

## Conclusion

This study revealed that higher insulin levels and higher degree of IR are significantly more likely to be present in newly diagnosed T2DM patients with higher BMI and WC. There was a significant positive correlation between BMI, WC and HOMA while a significant negative correlation with QUICKI and MCA in the cohort of patients with newly diagnosed T2DM in Sri Lanka. Accordingly, simple, low cost anthropometric measurements such as BMI and WC with the cut-off values of 24.91 kg/m^2^ and 81.5 cm respectively could be used as simple clinical markers to differentiate IR and ID in newly diagnosed patients with diabetes. Further, we highlight that young age and high TG/HDL-C ratio in patients with newly diagnosed T2DM are more likely to have high insulin level. Our findings could be useful in rational therapeutic decision making in the management of hyperglycemia in the newly diagnosed patients with T2DM in limited resource settings.
